# Impact of chronic urticaria on the quality of life of patients followed up at a university hospital *

**DOI:** 10.1590/abd1806-4841.20165071

**Published:** 2016

**Authors:** Gabriela Andrade Coelho Dias, Gisele Viana Pires, Solange Oliveira Rodrigues do Valle, Sérgio Duarte Dortas Júnior, Soloni Levy, Alfeu Tavares França, Ilaria Baiardini, Walter Giorgio Canonica

**Affiliations:** 1 Universidade do Estado do Rio de Janeiro (UERJ) – Rio de Janeiro (RJ), Brazil; 2 Universidade Federal do Rio de Janeiro (UFRJ) – Rio de Janeiro (RJ), Brazil; 3 Hospital São Zacarias – Rio de Janeiro (RJ), Brazil; 4 University of Genoa – Genoa, Italy

**Keywords:** Angioedema, Quality of Life, Questionnaires, Urticaria

## Abstract

**BACKGROUND:**

Chronic urticaria is a debilitating disease that considerably affects health-related quality of life, and the Chronic Urticaria Quality of Life Questionnaire is the only questionnaire specifically designed for its evaluation.

**OBJECTIVE:**

To evaluate the quality of life of patients with chronic urticaria, using the Brazilian Portuguese version of the Chronic Urticaria Quality of Life Questionnaire.

**METHODS:**

The Chronic Urticaria Quality of Life Questionnaire was self-administered in 112 chronic urticaria patients and disease activity was assessed through the Urticaria Activity Score. Clinical and socio-demographic characteristics of patients were studied, such as: age, sex, etiologic diagnosis of chronic urticaria, duration of disease and Urticaria Activity Score.

**RESULTS:**

The population studied was composed 85.72% of women with a mean age of 46 years (18-90), while the median disease duration period was 10 years (3 months-60 years). Regarding the etiologic diagnosis, 48.22% had chronic spontaneous urticaria; 22.32% associated with inducible urticaria, 28.57% with chronic autoimmune urticaria, and 23.21% had physical urticaria alone. Disease activity evaluated using the Urticaria Activity Score was 1.04 ± 1.61 (0-6). The total score for the Chronic Urticaria Quality of Life Questionnaire was 36 (0-100) and dimension I (sleep/mental status/eating) had a greater impact on quality of life. The items with the highest mean scores were nervousness and shame over lesions, while the items with the lowest scores were lip swelling and limitations on sporting activities.

**CONCLUSIONS:**

Chronic urticaria compromises patients' quality of life, mainly those with more severe disease or who are diagnosed with chronic autoimmune urticaria.

## INTRODUCTION

Chronic urticaria (CU) is a debilitating allergic skin disease, which affects 0.5 to 1% of the population and is characterized by erythematous, papulous and ichty lesions of a fluctuating nature that persist for over six weeks.^1^ It is highly complex in relation to its etiology and treatment is challenging, even for experts.

Quality of life (QoL) has become an important subject for society and especially for health professionals. It can be defined as the individual satisfaction or happiness with life in domains that the subject considers important. Several factors may affect a subject's well-being, such as work, housing and financial concerns. Health is but one of these factors. The expression "health-related quality of life" (HRQoL) was thus developed to refer to the disease's impact on and therapy in a patient's life, according to his/her perception. Hence, it is a subjective evaluation from the patient regarding the impact of health status on his/her full ability of living.^2^

In recent decades, the incorporation of patients' perceptions into decision-making over the handling of diseases has been an essential factor in improving the QoL of health assistance models. The incapacity of traditional clinical parameters to express what people feel and think has led to increased interest in the HRQoL field from the scientific community.^3^

CU interferes with subjective well-being and daily life; some patients' health status is comparable to that of coronary artery disease and severe asthma patients. It also causes inconvenience in family structures, compromising performance at work, school, and negatively impacting on leisure activities.^4,5^

Skin pruritus causes variable discomfort, as well as lesions, which, depending on their number and location, may harm an individual's physical appearance and social life.^6^

Sleep disorders such as insomnia, fatigue and drowsiness - due to pruritus or side effects from antihistamines - are frequently observed. Patients complain of recurrent pain syndromes, including tension headaches and fibromyalgia, while the prevalence of psychiatric disorders, like depression, hysteria, hypochondria and post-traumatic stress disorder is high.^7-9^

The degree to which quality of life is affected varies according to the chronic urticaria's etiology and severity. When CU is associated with delayed pressure urticaria, it affects quality of life more significantly than urticaria alone.^4^

HRQoL evaluation of patients with CU is essential for a better assessment of disease progression and treatment response. In 2005, Baiardini *et al.* developed and validated a specific questionnaire to evaluate the HRQoL in CU: the Chronic Urticaria Quality of Life Questionnaire (CU-Q_2_oL), which has been shown to, have satisfactory psychometric properties.^10^ The CU-Q_2_oL, originally written in Italian, was already successfully validated in Spanish, German, Polish, Turkish, Brazilian Portuguese, Korean, Greek and Persian.^11-18^

This study sought to evaluate the HRQoL in CU patients followed up at the outpatient chronic urticaria clinic of *Hospital Universitário Clementino Fraga Filho* (HUCFF) – *Universidade Federal do Rio de Janeiro* (UFRJ), who have participated in the transcultural validation process of the Brazilian Portuguese version of the CU-Q_2_oL, recently adapted and validated by our group^15^.

## METHODS

This was a cross-sectional study conducted in the same patient's sample evaluated in the Brazilian Portuguese cross-cultural adaptation of the CU-Q_2_oL study, published in 2011^15^. The study population comprised both male and female patients aged over 18, clinically diagnosed with chronic urticaria, characterized by the ocurrence of erythematous, papulous, pruriginous lesions, for a period of over six weeks, screened from July 2009 to August 2010 at the HUCFF urticaria outpatient clinic. The study excluded patients diagnosed with acute urticaria, contact urticaria, urticarial vasculitis, angioedema with no urticaria, patients unable to understand study terms and to give written consent, and patients with associated psychiatric comorbidities - 156 patients were evaluated and 44 were excluded. The study was approved by the Hospital Ethics Committee, and all patients provided written informed consent to participate.

The Brazilian version of CU-Q_2_oL includes 23 items that are divided into three dimensions: sleep/mental status/eating, pruritus/impact in daily activities, and limitations/appearance/edema.^19^

The questionnaire refers to the preceding two weeks, and the patients indicate the intensity of each item separately, on a 5-point Likert scale, ranging from 1 = "not at all" to 5 = "very much". A score is calculated for each dimension, then a total index is calculated for all the dimensions. The score ranges from 0 to 100. The higher the score, the worse the patient's perception of his/her quality of life is.

Urticaria severity was evaluated using the score proposed by Zurberbier *et al.* (2001); and disease severity was assessed via the UAS – Urticaria Activity Score. The score evaluates the number of lesions and pruritus intensity. The sum of scores obtained by evaluating urticaria and pruritus ranges from 0 to 6, where 0 corresponds to controlled disease, while 6 corresponds to high intensity disease.^20^

The following clinical and socio-demographic characteristics of the patients were studied, such as: age (18-40 years, 41-60 years, > 60 years), sex, etiologic diagnosis of chronic urticaria (chronic spontaneous urticaria, chronic autoimmune urticaria and inducible urticaria), time to disease progression (< and > 5 years) and UAS (group 1: scores 0 and 1; group 2: scores 2, 3 and 4; group 3: scores 5 and 6). Moreover, the ANOVA analysis was used to evaluate the differences between the groups.

Statistical analysis was performed through SPSS 17.0 and Stata 8.0. A descriptive statistical analysis was used in the clinical and demographic characterization of the patients studied, and an analysis of variance (ANOVA) was applied to compare three or four independent means - p < 0.05 was considered statistically significant.

This study and the respective informed consent form were approved by the Hospital Ethics Committee.

## RESULTS

The population studied consisted of 96 female patients (85.72%) and 16 male patients (14,28%), with a mean age of 46 years (18-90) and a mean family income of R$1,828.66. In this sample, 58.03% of patients were married, 37.5% had not completed elementary school, and 8.03% had completed higher education, 49.11% were employed, 27.68% were homemakers, and 13.40% were retired (Table 1).

**Table 1:** Socio-demographic characteristics

Variable	n = 112
Sex (n/%)	
Male	16 (14.28)
Female	96 (85.72)
**Age (variation)**	46 years old (18-90)
**Marital status (n/%)**	
Married	65 (58.03)
Single	29 (25.90)
Divorced	7 (6.25)
Widow/widower	11 (9.82)
**Scholarship (n/%)**	
Uncompleted elementary school	42 (37.50)
Elementary school	19 (16.97)
High school	37 (33.04)
Higher education	9 (8.03)
Post-graduate	5 (4.46)
**Occupation (n/%)**	
Employee	55 (49.11)
Homemaker	31 (27.68)
Retired	15 (13.40)
Student	5 (4.46)
Unemployed	6 (5.35)
Income (standard deviation – SD)	R$1,828.66 – R$2,015.86

The mean time to disease progression was 10 years (3 months – 60 years), and the follow-up time at hospital was 4 years. Regarding the etiologic diagnosis, 48.21% of patients had chronic spontaneous urticaria (CSU), 22.32% associated with physical urticaria, 28.57% had chronic autoimmune urticaria (CAU) and 23.21% had inducible urticaria alone. Furthermore, 59.82% of patients required continuous treatment with antihistamines (Table 2).

**Table 2:** Sample clinical characteristics

Variable	n = 112
Time to disease progression	10.6 years (3 months to 60
(variation/SD)	years/10.6 years)
Time of follow-up on the job	4 years (0 to 25 years/4.4 years)
(variation/SD)	
Type of urticaria (n/%)	
Chronic spontaneous urticaria	
Alone	29 (25.90)
Associated with inducible	25 (22.32)
urticaria	
Autoimmune urticaria	
Alone	8 (7.15)
Associated with inducible	24 (21.42)
urticaria	
Inducible urticaria alone	26 (23.21)
Continuous use of drugs (n/%)	67 (59.82)

Disease activity evaluated by UAS score was 1.04 ± 1.61 (0-6). Of the 112 patients enrolled in the study, 8 (7%) did not answer at least one question from the CU-Q_2_oL. The item with the highest ratio of blank answers was item 4 (lip swelling). The mean total score for the questionnaire was 36, while dimension I had the highest score; however, the score values were homogeneous for all three dimensions (Table 3).

**Table 3:** CU-Q2oL dimension scores

Dimension	Item (0-100)	Mean score	SD
Total score		36	22
I – Sleep/mental status/eating	10,11,12,13,14,15,16,17	39.9	24.7
II – Pruritus/impact on daily activities	1,2,5,6,7,8,9,22	34.4	26.4
III – Edema/limitations/appearance	3,4,18,19,20,21,23	34.8	24.8

The items with the highest mean scores were 15 (nervousness), followed by 18 (shame over lesions) and 1 (pruritus). The lowest scores were item 4 (lip swelling) and 22 (limtations on sporting activity) (Table 4).

**Table 4:** Item scores

Item		Mean Score (0-100)
1	Pruritus	49.0
2	Wheals	37.5
3	Eyes swelling	20.8
4	Lip swelling	12.6
5	Work	40.5
6	Physical activities	25.1
7	Sleep	41.5
8	Free time	29.5
9	Social relationships	31.2
10	Eating	28.2
11	Falling asleep	36
12	Waking up at night	44.3
13	Tired	43.3
14	Concentration	37.5
15	Nervousness	55.0
16	Bad Mood	39.3
17	Limits foods	35
18	Embarrassed by signs	52.8
19	Embarrassed in public	36.8
20	Cosmetics	39.5
21	Limits clothes	37.8
22	Sports	17.5
23	Medication side effects	41.8

Seventy-five percent of the patients were not doing sporting activities and only 16% indicated chronic urticaria as the reason for this. Of the 28 patients who played sports, 42.8% mentioned that urticaria interfered a little, somewhat, or too much in sporting activity-related quality of life. Of the 13 patients who were not practicing sports due to urticaria, 69% reported that urticaria affected much or very much.

ANOVA analysis showed that patients aged 41-60 years were patients with autoimmune urticaria were more impacted in dimension III, compared with patients suffering from chronic spontaneous urticaria and inducible urticaria alone. Women were more affected in all dimensions but not in a statistically significant manner. Patients with higher severity scores (group 3) experienced a greater impact on quality of life in the total score, and in dimensions II and III (Table 5).

**Table 5:** Distribution of scores and clinical characteristics

	Total score 0-100/n	Sleep / mental status / eating (0-100)	Pruritus / impact on daily activities (0-100)	Edema / limitations / appearance (0-100)
**Sex**				
Female	36.30 (96)	40.42	41.63	35.84
Male	36.06 (16)	36.94	33.30	28.88
**Age**				
18-40 y	33.03 (36)	32.50^a^	35.58	31.53
41-60 y	41.07 (56)	48.32^a^	37.64	37.50
>60 y	28.65 (20)	29.75^a^	23.70	33.40
**Type**				
Spontaneous	38.53(54)	40.53	38.79	36.66^b^
Autoimmune	40.47 (32)	46.16	34.81	41.28^b^
Inducible alone	27.50 (26)	31.69	26.65	24.31^b^
**Disease duration**				
< 5 years	35.66 (47)	39.09	36.04	31.66
> 5 years	36.71 (65)	40.52	33.06	37.15
**Groups UAS**				
1	32.06a (85)	37.34	28.06^c^	31.29^d^
2	46.53a (19)	48.16	47.47^c^	44.37^d^
3	56.63a (8)	47.75	72.00^c^	50.00^d^

a p=0,001,

b p=0,03,

c p<0,0001,

d p=0,02

## DISCUSSION

CU seriously compromises the HRQoL of patients due to debilitating and uncomfortable symptoms that may last for years.^5^

In addition to classic symptoms, like pruritus and papules, other factors are more relevant for patients with chronic urticaria, such as unpredictability of flares, sleep disorders, fatigue, drug-related side effects, and physical appearance. Thus, merely evaluating urticaria progress by counting lesions and measuring pruritus intensity is insufficient. A holistic evaluation of the patient is required for a better understanding of disease impact. In 2010, the Global Allergy and Asthma European Network (GA_2_LEN) study group recommended evaluating the Patient-Reported Outcomes (PRO) and HRQoL in clinical trials on allergy, recognizing the importance of better knowledge of subjective evaluation of patients in relation to health status factors.^21^

Most studies on quality of life concerning chronic urticaria have used generic questionnaires or those for dermatological diseases, such as the DLQI (Dermatology Life Quality Index), which was validated as a useful instrument to evaluate the HRQoL of patients with chronic urticaria.^22^ However, the DLQI can be used for any dermatological disease, since it was not developed specifically chronic urticaria patients and may not measure important factors concerning them. CU-Q_2_oL is a valid and specific instrument to concerning them. CU-Q_2_oL is a valid and specific instrument to evaluate the HRQoL. It is easily applied and requires five minutes to be completed by the patient.^10^ This study aimed to evaluate the impact of chronic urticaria on the quality of life of patients followed up at a university outclinic, to determine which aspects of quality of life affected these patients the most.

The population evaluated in this study showed a high prevalence of chronic urticaria in women (85.72%). Epidemiological studies demonstrate that occurrence is twice as high in female patients.^23^ A population investigation conducted in Germany demonstrated that in a sample of 4,093 people, 1.8% had CU, of which 70.3% were women, while the sample from the CU-Q_2_oL original study included 61.84%.^10,24^ It is frequently noted that women are more affected by chronic urticaria and other autoimmune diseases.

In most studies, the peak age of CU occurrence is between 20 and 40 years.^25^ Thus, patients are mainly affected during their working life years and are more prone to absenteeism and decreased productivity because of illness and its treatment. A Spanish study observed that the mean age of patients was 35.75±18.9 years and this study population had a mean age of 46 years (18–70 years).^26^

Patients had a high educational level compared with the overall population, with 45% having at least completed high school, similarly to the findings of the original study (51.31%).^10^ The average income was R$1,828.66, while the average income of Brazilian families in 2010 was R$1,292.00.^27^ Many diseases have a prevalence standard, which is dependent on socio-economic and educational levels. With respect to urticaria, there is little data available on this issue. Several studies were not able to show a difference in the prevalence of urticaria in terms of educational level, occupation, income, residence location, and ethnic origin.^24,26^

The mean time to disease progression was 10.6 years (3 months–60 years). In the Baiardini *et al.* study, the time was shorter, around 1 year and 9 months (SD: 27.32 months).^10^ Gaig *et al.* found that 50% of CU patients were asymptomatic in three months, and 80% in 12 months. However, 11% were affected for over five years.^28^ In most cases, chronic urticaria typically remits after 1-5 years, though 10-20% of cases may last 5-10 years and some can persist for up to 50 years. Patients with severe urticaria at diagnosis usually experience longer durations. In our population, 61% of patients presented the disease for over five years. The authors believe that the high frequency of long-lasting cases in these series is due to the institution being a reference center, which tends to recruit severe and refractory cases.

Regarding the etiology of chronic urticaria, just like in the medical literature, a higher prevalence of CSU (48.21%) was observed, followed by CAU (28.56%), and physical urticaria alone (23.21%).^1^ Nevertheless, when all patients with inducible urticaria (alone and associated) were considered, a prevalence of 66.95% was found. In the Spanish study, patients experienced chronic spontaneous urticaria in 68% of cases, and physical urticaria in 60%.^11^ About half of the study population did not have an etiologic diagnosis despite having undergone a complete research protocol with challenge tests for physical urticaria and an autologous serum test. Affected patients with chronic spontaneous urticaria suffer deep frustration because of the uncertainty about the cause of their disease, which reduces their quality of life.

The first application of the Brazilian Portuguese version of *CU-Q2oL* showed that it is a useful tool to evaluate the disease more specifically.

The dimension score was not elevated, ranging from 34.4 to 39.9 on a scale of 0 to 100. This is because the study population was very heterogeneous, encompassing patients with different types of urticaria at various stages of disease progression. The mean UAS score was low (1.04 ± 1.61 [0-6]), as 66 patients (58%) were asymptomatic on evaluation day.

Dimension I (sleep/mental state/eating) revealed a greater the disease entails sleep disorders, causing significant damage to mental health, as well as chronic fatigue, loss of professional productivity and commitment to their personal and social lives. Previous studies have reported that chronic urticaria has a significant impact on quality of life, especially as regards sleep and energy, which is consistent with our results.^4,29,30^

Question 15, "nervousness", had the highest score (55). This finding confirms the high impact of chronic urticaria on the patients' mental health. Pasaoglu *et al.* demonstrated that patients with chronic spontaneous urticaria had a higher prevalence of depression, hysteria, hypochondria, and conflicts with their social environment.^8^

The questions "embarrassed by signs" and "pruritus" also revealed a great impact on quality of life, as the first question assesses patients feelings and the second, the main and most troublesome symptom of this disease, which interferes with daily activities and mental health.

The question on "lip swelling" had the lowest score, like in the Spanish study.^11^ Sporting activities were not regarded as relevant by the patients either, probably because 75% of the patients involved in this study were not practicing sports and urticaria was the reason for only 13% of them. The precursor O´Donnel study showed that 45% of CU patients reported limitations for running.^4^

Regarding gender, women were expected to be more affected than men.^12,31^ Women likely have more sensitive skin and mental awareness toward the symptoms of hives and are more influenced by changes in appearance. In fact, a greater impact was found in all dimensions for women, though there was no statistical significance.

In relation to age, patients aged between 41 and 60 years had a higher impact in dimension I. This age group includes adults at the peak of their professional lives, and disturbances in sleep and mental state limit their working lives.

Variance analysis of *CU-Q_2_oL* results showed that patients with autoimmune urticaria (CAU) had the worst quality of life in dimension III (edema, appearance, and limitations). Several studies evaluating disease severity have concluded that patients with positive autologous serum skin tests were more affected than those with negative tests.^23^ A multicenter study published in 2009 revealed the highest disease severity and worst quality of life measured by DLQI in patients with positive autologous serum skin tests.^32^

Severity assessment (UAS) demonstrated a strong correlation with the quality of life impact. Patients with more severe disease showed greater degree of impairment, especially in dimension II (itching/impact on daily activities).

In Brazil, there are few studies evaluating HRQoL in patients with chronic urticaria. However, it was shown that these patients underwent an important change in QoL; the most affected domains were food restrictions, emotional changes and quality of sleep.^33^ A study published in 2011 using DLQI and SF-36, a generic instrument to evaluate quality of life, demonstrated greater impairment in: women, patients aged up to 30, those experiencing their first visit, patients with a high level of education, those who had the disease for up to 1 year, and angioedema patients.^31^ In another Brazilian study published in 2011, the patients had a mean score of 13.5 in the DLQI (0-30), the presence of angioedema was associated with higher scores (14.3; p < 0.01); women were more limited with respect to clothing and men with respect to work and study (p < 0.05).^34^ In these studies, the presence of angioedema and being female were associated with a worse quality of life. This study highlights a tendency toward a worse quality of life in women. The difference between patients with and without angioedema was not assessed. There is a need for further studies to define the predictive factors that affect quality of life in the Brazilian population.

## CONCLUSION

Chronic urticaria seriously compromises the quality of life of patients due to its debilitating symptoms that can last for years. In this study, a major impairment was observed in patients with the highest severity and in those diagnosed with autoimmune urticaria. An evaluation of quality of life is fundamental to better assess disease progression and treatment efficacy, as per recommended by the GA_2_LEN. In the future, the Brazilian Portuguese version of the CU-Q_2_oL may enable multicenter studies to be performed, in addition to promoting an overall understanding of the impact of chronic urticaria.
